# Supplementary material for: Mechanisms governing the pioneering and redistribution capabilities of the non-classical pioneer PU.1
Source: Nat Commun. 2020 Jan 21;11:402. doi: 10.1038/s41467-019-13960-2 (PMC6972792; doi:10.1038/s41467-019-13960-2)
Supplement: Supplementary file 7 — Source data [file 41467_2019_13960_MOESM7_ESM.zip › Source_Data/FigureS2/FigureS2B_MotifScanOutput/KmeansCluster12/homerResults/motif12.similar.html]

motif12

## Information for motif12

G
A
C
T
A
C
T
G
C
A
G
T
T
C
A
G
A
C
T
G
C
T
A
G
G
T
C
A
C
T
A
G
C
A
T
G
  
Reverse Opposite:  

G
T
A
C
G
A
T
C
C
A
G
T
A
G
T
C
G
T
A
C
A
G
T
C
G
T
C
A
A
G
T
C
C
T
G
A
  

|  |  |
| --- | --- |
| p-value: | 1e-30 |
| log p-value: | -6.979e+01 |
| Information Content per bp: | 1.719 |
| Number of Target Sequences with motif | 545.0 |
| Percentage of Target Sequences with motif | 30.33% |
| Number of Background Sequences with motif | 8627.0 |
| Percentage of Background Sequences with motif | 18.75% |
| Average Position of motif in Targets | 98.6 +/- 58.0bp |
| Average Position of motif in Background | 99.5 +/- 65.2bp |
| Strand Bias (log2 ratio + to - strand density) | 0.2 |
| Multiplicity (# of sites on avg that occur together) | 1.23 |
| Motif File: | file (matrix) reverse opposite |

### Similar de novo motifs found

|  |  |  |  |  |  |  |  |
| --- | --- | --- | --- | --- | --- | --- | --- |
| Rank | Match Score | Redundant Motif | P-value | log P-value | % of Targets | % of Background | Motif file |
| 1 | 0.793 | A C T G C G A T A C T G C A T G T C A G A C T G C T A G T C A G C T A G C T A G | 1e-28 | -66.680094 | 32.44% | 20.78% | motif file (matrix) |
| 2 | 0.844 | G A C T A C T G C G A T A C T G C T A G T C A G A G T C C A T G | 1e-21 | -49.234819 | 18.59% | 10.75% | motif file (matrix) |
| 3 | 0.756 | A G T C A T G C A G T C G A T C T G A C G T A C A T G C A T G C | 1e-14 | -34.175606 | 18.20% | 11.64% | motif file (matrix) |
